# Health care professionals’ perceptions about atrial fibrillation care in the Brazilian public primary care system: a mixed-methods study

**DOI:** 10.1186/s12872-022-02927-9

**Published:** 2022-12-22

**Authors:** Elisabete Paschoal, Tiffany E. Gooden, Rodrigo D. Olmos, Paulo A. Lotufo, Isabela M. Benseñor, Semira Manaseki-Holland, Gregory Y. H. Lip, G. Neil Thomas, Kate Jolly, Emma Lancashire, Deirdre A. Lane, Sheila Greenfield, Alessandra C. Goulart, on behalf of NIHR Global Health Research Group on Atrial Fibrillation Management, Ajini Arasalingam, Abi Beane, Peter Brocklehurst, Kar Keung Cheng, Wahbi El-Bouri, Mei Feng, Yutao Guo, Mahesan Guruparan, Gustavo Gusso, Rashan Haniffa, Lindsey Humphreys, Sue Jowett, Chamira Kodippily, Balachandran Kumarendran, Xuewen Li, Yan-guang Li, Trudie Lobban, David Moore, Krishnarajah Nirantharakumar, Paskaran Pirasanth, Uruthirakumar Powsiga, Carla Romagnolli, Itamar S. Santos, Alena Shantsila, Vethanayagan Antony Sheron, Kanesamoorthy Shribavan, Isabelle Szmigin, Kumaran Subaschandren, Rajendra Surenthirakumaran, Meihui Tai, Bamini Thavarajah, Timo Toippa, Ana C. Varella, Hao Wang, Jingya Wang, Hui Zhang, Jiaoyue Zhong

**Affiliations:** 1 Center for Clinical and Epidemiological Research, Hospital Universitário, Universidade de Sao Paulo, Sao Paulo, Brazil; 2 Institute for Applied Health Research, University of Birmingham, Birmingham, UK; 3 School of Medicine, Universidade de Sao Paulo, Sao Paulo, Brazil; 4 Liverpool Centre for Cardiovascular Science, University of Liverpool and Liverpool Heart & Chest Hospital, Liverpool, UK; 5 Department of Clinical Medicine, Aalborg University, Aalborg, Denmark

**Keywords:** Atrial fibrillation, Mixed-methods, Healthcare professionals, Qualitative, Questionnaire, Primary care, Brazil, LMIC

## Abstract

**Background:**

Atrial fibrillation (AF) negatively impacts health systems worldwide. We aimed to capture perceptions of and barriers and facilitators for AF care in Brazilian primary care units (PCUs) from the perspective of healthcare professionals (HCPs).

**Methods:**

This mixed-methods, cross-sectional study utilised an exploratory sequential design, beginning with the quantitative data collection (up to 18 closed questions) immediately followed by a semi-structured interview. HCPs were recruited from 11 PCUs in the Sao Paulo region and included managers, physicians, pharmacists, nurses and community health agents. Descriptive statistics were used to present findings from the quantitative questionnaire and inductive analysis was used to identify themes from the qualitative data.

**Results:**

One hundred seven HCPs were interviewed between September 2019 and May 2020. Three main themes were identified that encapsulated barriers and facilitators to AF care: access to care (appointments, equipment/tests and medication), HCP and patient roles (HCP/patient relationship and patient adherence) and the role of the organisation/system (infrastructure, training and protocols/guidelines). Findings from the qualitative analysis reinforced the quantitative findings, including a lack of AF-specific training for HCPs, protocols/guidelines on AF management, INR tests in the PCUs, patient knowledge of AF management and novel oral anticoagulants (NOACs) as key barriers to optimal AF care.

**Conclusions:**

Development and implementation of AF-specific training for PCU HCPs are needed in Brazil, along with evidence-based protocols and guidelines, educational programmes for patients, better access to INR tests for patients taking warfarin and availability of NOACs.

**Supplementary Information:**

The online version contains supplementary material available at 10.1186/s12872-022-02927-9.

## Background

Atrial fibrillation (AF) has a huge impact on public health systems worldwide [1]. Although high-income countries still experience high prevalence, incidence, disability, and mortality rates related to AF, a greater burden of disability related to AF, particularly stroke, is concentrated in low- and middle-income countries (LMICs) [2, 3]. AF is responsible for about 20% of ischaemic strokes, and this rate increases with age [4]. Adequate AF management is still a challenge in LMICs, including Brazil which has one of the highest rates of stroke in Latin America [5] and, where there is an underuse of oral anticoagulation (OAC) even in individuals at high risk of stroke [6, 7]. Indeed, a recent systematic review reported that only 45% of individuals with CHA_2_DS_2_-VASc scores ≥ 2 were receiving OAC therapy in South American countries [6] and a long-term stroke cohort reported OAC use for about 10% among AF stroke patients [7].

Recent qualitative studies conducted in high-income countries, mostly performed with physicians and patients, revealed that OAC therapy is hampered by many factors [8–14]. Doctors have reported uncertainty about the use of OAC treatment mainly due to patients' knowledge gaps and misconceptions, fears of bleeding and the need for individualised decision making [8–10]. From the patient's perspective, issues such as bleeding risk, need for frequent monitoring (for vitamin K antagonists) and medication costs have been the main concerns reported [8–14].

Despite the recognised burden of AF worldwide, no previous studies have focused on the AF care pathway in LMICs, which have the highest burden of stroke [15]. Thus, we aimed to identify general perceptions, problems, facilitators for and barriers to the optimal management of AF from the perspective of healthcare professionals (HCPs) who regularly work in the primary care units (PCUs) in a low-income area located in the city of Sao Paulo, Brazil.

The present study was conceptualised by the National Institute for Health Research Global Research Group on AF Management (NIHR Global AF Reach) at the University of Birmingham and University of Liverpool [16] that has developed a partnership with LMICs and works with disadvantaged populations in China, Brazil, and Sri Lanka to improve the management of AF in public healthcare networks.

## Methods

This research was approved by the local ethics committee from the Hospital Universitario of the University of Sao Paulo (Reference: 94,732,318.6.0000.0076) and the National Ethical Committee for International Research in Brazil (Reference: 3.301.920) with subsequent review and approval by the University of Birmingham Ethics Committee (Reference: ERN_19-1898). All participants gave informed written consent and provided their signature through an electronic form.

### Study design

This mixed-methods cross-sectional study utilised an exploratory sequential design. It started with the quantitative data collection immediately followed by a semi-structured interview to identify HCPs’ knowledge and experiences of AF care and management and any barriers or facilitators for optimal AF care in PCUs [17, 18].

### Study setting and study participants

**Fig. 1:**
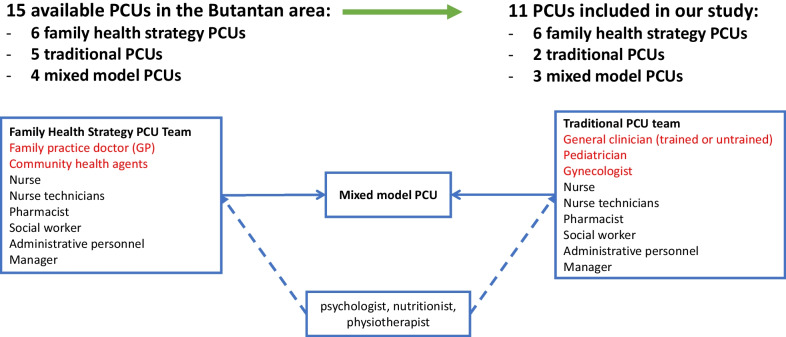
Structure of primary care units in the Butantan area of Sao Paulo, Brazil. Dotted arrows represent healthcare professionals that are sometimes available. Healthcare professionals in red represent the difference between the models

This study was conducted in a socioeconomically deprived area in the Butantan district in the city of Sao Paulo, Brazil. Universal healthcare is well established in Brazil with all healthcare visits, medications, and treatments available free of charge at the point of care [19]. The estimated prevalence of AF in the Butantan area is 2.4% in older adults [20]. There are 15 PCUs in Butantan which covers approximately 500,000 people. Eleven PCUs were included in this study due to their connection with the University of Sao Paulo (i.e. they include medical fellowships and medical students) where the research team was based. There are three different models of PCUs in the region of Butantan: Model 1, the family health strategy (FHS) PCU which comprises 4 to 10 key team members with at most one family practice doctor (i.e. GP), one nurse, two nurse technicians and six community health agents; Model 2, the traditional PCU varies in size and includes at least one general clinician (who may be without specialty training i.e. only medical school training), paediatrician, gynaecologist, nurse and nurse technician; and Model 3, the mixed model PCU has FHS and traditional teams working in the same unit. All PCU models have a manager, administrative staff and at least one pharmacist and social worker. Some PCUs also have psychologists, nutritionists and physiotherapists. (Fig. 1). Six FHS PCUs, two traditional PCUs and three mixed model PCUs were included in our study. Managers from each PCU were contacted by phone and asked to identify eligible HCPs that could take part (i.e. HCPs with experience caring for AF patients); they then sent the researchers a list of eligible HCPs to contact for participation. Each HCP from the list was invited to take part in the study by phone. General responsibilities of each HCP are listed in Table 1.

**Table 1:** Roles and responsibilities of included healthcare professionals in Sao Paulo, Brazil

Healthcare professionals	Responsibilities
Manager	Plans, manages and organises the work process; coordinates home visits and care in the community, integrates the units with other public health services
**Family Health Strategy Primary Care Unit**	
Family practice doctors (general practitioner)	Cares for 3,500 to 4,500 people; undertakes patient appointments, health-specific community group events and home visits
Nurse	Cares for 3,500 to 4,500 people; undertakes patient appointments, health-specific community group events and home visits; supervises the family health team
Nurse technicians	Manage medications (check adherence, pill count and explain side effects); takes patients’ blood pressure measurement and other procedures; guides on how to take prescriptions such as insulin and how to use home tests such as a glucometer
Community health agents	Visits all patients and their family members at least 1 time per month; checks adherence to medication, vaccination status and for any issues in the home that may impact their health
**Traditional Primary Care Unit**	
General clinicians (recently graduated doctors)	Undertakes mostly patient appointments and some health-specific group events
Pharmacist	Checks prescriptions; delivers medication; guides on how to take medications and its side effects
Nurse	Cares for 3,500 to 4,500 people; undertakes patient appointments and health-specific group events
Nurse technicians	Manage medications (check adherence, pill count and explain side effects); takes patients’ blood pressure measurement and other procedures; teaches patients how to take prescriptions such as insulin and how to use home tests such as a glucometer

### Data collection

The data collection tools were developed in collaboration between researchers at the Universities of Birmingham, Liverpool and Sao Paulo. A trained anthropologist (EP) carried out data collection face-to-face or by phone. We gave participants as much time as necessary to answer the questions; however, for the quantitative questions, it took most participants up to 5 min to complete and many found it easy and quick to answer the qualitative questions. The total time it took participants to complete the questionnaire and semi-structured interview was between 15 and 30 min. All interviews were audio recorded and transcribed verbatim in Portuguese then translated into English then back-translated to check for accuracy. The interviewer took complementary field notes and added them to the final transcripts.

The quantitative questionnaire comprised up to 18 closed questions including five multiple choice questions (Additional file 1). These questions examined HCPs’ knowledge and experience of AF management and care, particularly OAC therapy and anticoagulation control. The semi-structured interview comprised five questions to elicit information regarding the care pathway and any barriers or facilitators related to patient-HCP relationship and the care and monitoring of AF patients. The responses to the quantitative questions were used as the reference point to elaborate on barriers and facilitators asked about within the semi-structured interviews. The initial five questions were 1) Who are the patients with AF that you follow? 2) How is AF diagnosed and followed-up? 3) In your opinion, what are the positive and negative points around caring for and monitoring AF patients? 4) How is the interaction between AF patients and you and other team members at this PCU? and 5) In your opinion, how could the interaction between AF patients and you and the other team members be improved at this PCU?.

### Data analysis

Descriptive statistics were used to present the findings for quantitative data, presenting frequencies and percentages overall and by HCP profession. HCPs’ experiences of AF and the care pathway were analysed using data from the first two open-ended questions (“Who are the patients with AF that you follow?” and “How is AF diagnosed and followed-up?”). The qualitative data from the remaining questions (questions three to five) were grouped and synthesised together for identifying barriers and facilitators for optimal management of AF. The qualitative data were analysed by EP, the same person who conducted the data collection, using a process of coding line-by-line, reviewing and regrouping. Themes and sub-themes were derived from the data through an iterative process of developing themes and sub-themes [21, 22], consulting with the remaining team from Brazil and UK including qualitative experts at the Universities of Birmingham and Liverpool, then revisiting the data for further development of themes and sub-themes.

We cross-examined information from the quantitative questionnaire and semi-structured interviews to provide an in-depth narrative about HCPs’ experience of AF care and management in Brazilian PCUs [21, 22].

## Results

Data were collected between September 2019 and May 2020. From the 200 HCPs identified to take part in this study, 36 were unavailable due to the COVID-19 pandemic and 57 did not have experience of caring for AF patients and were subsequently excluded. The remaining 107 HCPs were invited and agreed to take part: 9 managers, 22 physicians (13 family practice doctors and 9 general clinicians), 16 nurses, 18 nurse technicians, 29 community health agents and 13 pharmacists. Eighty-three percent (*n* = 89) of HCPs were female with a mean age of 37.9 years (standard deviation: 8.5 years) (Additional file 2). Data were collected face-to-face for 101 HCPs (94%) and by telephone for 6 (6%).

### Results to closed questions

Eight of the nine managers reported no specific AF training for their staff; however, more than half (*n* = 5/9) said their staff had asked for support regarding AF management and two reported that staff had requested support with prescribing anticoagulants. Indeed, none of the family practice doctors or community health agents reported receiving specific training for treating AF whilst working in the PCU (Additional file 3). Similarly, most of the general clinicians (*n* = 7/9), nurses (*n* = 13/16), nurse technicians (*n* = 15/18) and pharmacists (*n* = 12/13) said they had not received specific training for AF treatment whilst working at the PCU.

When HCPs were asked if they followed AF-specific guidelines, less than half of the family practice doctors (*n* = 6/13), general clinicians (*n* = 3/9), nurses (*n* = 4/16), nurse technicians (*n* = 6/18), pharmacists (*n* = 4/13) and community health agents (*n* = 4/29) reported that they did (Additional file 3). However, more HCPs said they follow a risk scale for deciding on AF treatment: nine family practice doctors (69%), four general clinicians (44%), eight nurses (50%), seven nurse technicians (39%), four pharmacists (31%) and seven community health agents (24%).

Regarding AF diagnosis, all family practice doctors (*n* = 13/13), nurses (*n* = 16/16) and nurse technicians (*n* = 18/18) said their AF patients had an existing ECG confirming their diagnosis; most general clinicians (*n* = 8/9) and community health agents (*n* = 24/29) concurred (Additional file 3). Most family practice doctors (*n* = 12/13), general clinicians (*n* = 8/9), nurses (*n* = 11/16) and pharmacists (*n* = 11/13) reported that warfarin was the most prescribed OAC for their AF patients, whereas fewer nurse technicians (*n* = 7/18) and community health agents (*n* = 17/29) said warfarin was prescribed. In addition to warfarin, aspirin was commonly mentioned as medication that AF patients take (6/13 family practice doctors, 4/9 general clinicians, 12/16 nurses, 10/18 nurse technicians, 5/13 pharmacists and 19/29 community health agents). None of the pharmacists (*n* = 0/13) said that non-vitamin K antagonist oral anticoagulants (NOACs) are prescribed to AF patients. Conversely, few family practice doctors (*n* = 3/13), nurses (*n* = 3/16), nurse technicians (*n* = 5/18) and community health agents (*n* = 1/29) but more of the general clinicians (*n* = 5/9) reported that NOACs are prescribed.

All general clinicians (*n* = 8/8) and nurse technicians (*n* = 7/7) and most of the family practice doctors (*n* = 11/12), nurses (*n* = 9/11) and community health agents (*n* = 13/17) said they advise their AF patients on warfarin to take INR (international normalised ratio) tests in a secondary care facility (Additional file 4). Only four of the 11 pharmacists managing AF patients on warfarin said they referred them to secondary care for INR tests. When given a list of options relating to barriers in monitoring patients with AF under warfarin use, more than half of all HCPs stated that one of the main barriers was difficulty in patient understanding on how to take the medication (*n* = 41/66). More than half of all HCPs also felt that fear of severe bleeding (*n* = 38/66), interaction of warfarin with diet (*n* = 35/66), interaction of warfarin with other drugs (*n* = 34/66) were challenges in monitoring warfarin use. Difficulty performing INR tests, delays in test results and low patient adherence to treatment were additional challenges selected by nearly half of all HCPs (*n* = 32/66; *n* = 27/66; *n* = 28/66, respectively). Only one HCP said patients not bringing their anticoagulation control card was a challenge and 14 HCPs said difficulty of obtaining medications was an issue.

### Results of open questions

#### HCPs’ impression of the profile of AF patients that attend PCUs

HCPs had distinct views about the sociodemographic characteristics and lifestyle habits of their AF patients (Additional file 5). They described their patients as being elderly, male, having unhealthy lifestyle habits such as sedentarism, smoking, a regular diet with high consumption of fat and carbohydrates and having multi-morbidities.

“They are old men with comorbidities who already had a heart attack.” General clinician (traditional PCU)

“They are smokers, sedentary.” Nurse (traditional PCU)

#### HCPs’ description of the AF pathway of care

For diagnosing AF, HCPs stated that AF was identified through routine consultation or if the patient arrived feeling unwell. HCPs also claimed that patients are already diagnosed with AF before arriving for care or it was picked up on during home visits.

*“Some patients arrive feeling sick, so they see the doctor, we do the electro[cardiogram] and they are referred to the cardiologist.” Nurse (FHS PCU)*

*“Patients are identified at home during the monthly visits.” Nurse (FHS PCU)*

HCPs stated that patients return for follow-up care between 1 to 2 weeks before stabilisation and 1 to 6 months after stabilisation. It was clear from HCPs’ statements that the monitoring of AF is largely dependent on communication and referrals between PCUs and the hospitals. HCPs declared that they conduct electrocardiograms and provide advice on and adjustments to medication whereas cardiologists in the hospital perform INR tests, review the electrocardiogram results, and sometimes advise the doctors at PCUs on treatment.

“The doctor performs the electrocardiogram, forwards the patient to the cardiologist and then they start the anticoagulant together.” Nurse technician (traditional PCU)

“The patients who are diagnosed in the unit, we ask for an electrocardiogram, forward it to the cardiologist.” Family physician (FHS PCU)

#### HCPs’ perspective of barriers and facilitators for AF care in PCUs

Regarding barriers and facilitators for the care and management of AF patients, three main themes arose: access to care, HCP and patient roles and the role of the organisation/healthcare system. Three sub-themes emerged for access to care: access to appointments, equipment/tests, and medication. Facilitators included availability of consultations/appointments with the doctors, electrocardiograms, and warfarin at PCUs:

*“As soon as the doctor diagnoses the presence of AF, he asks for the electrocardiogram and the patient does it right away here in the unit.” Manager (FHS PCU)*

*“The necessary medications are available. Warfarin is never lacking.” Pharmacist (mixed model PCU)*

Barriers to access to care included a lack of INR tests in the PCUs, modern drugs and appointments to see a cardiologist along with challenges in traveling to see the cardiologist:

*“It should have INR collection in the [primary care] unit itself because patients have financial and locomotion difficulties to go to the Peri-Peri [secondary care facility].” Nurse technician (mixed model PCU)*

*“We should have better drug options, like NOACs [novel oral anticoagulants], which would make it much easier, as there is no need to control with lab tests and consultations with the specialist.” Family physician (mixed model PCU)*

Regarding the HCP and patient role for AF care, two sub-themes were identified: HCPs’ relationship with, and dedication to, their patients and patients’ adherence to HCPs’ advice/instructions. The former was determined as a facilitator given the good relationship and interaction between HCPs and patients along with HCPs’ commitment to their patient’s health and wellbeing. From the HCPs’ perspective, most patients were adherent to the information they provided them with (a facilitator), but some patients were not (a barrier).

“We have well-prepared teams, the professionals are close to the patients, they have a good relationship with the patients and their families.” Nurse technician (mixed model PCU)

“Patients usually follow the instructions from their doctors to take the medication correctly.” Family physician (mixed model PCU)

“Some patients are more resistant and do not accept the medications.” Community health agent (mixed model PCU)

The final theme referred to organisational/system roles which highlighted barriers but no facilitators. The lack of private space, specific training and protocols or guidelines on AF management were consistently mentioned. There was also mention of delays to INR test results.

“There is a lack of adequate physical space for consultations and specific training in AF care.” Nurse (traditional PCU)

“The INR exam results take more than 1 week to arrive! We would need to have the results on the same day!” Family physician (FHS PCU)

“There is a lack of training, and protocols for AF care.” Nurse technician (FHS PCU)

## Discussion

This mixed-methods study sought to understand facilitators and barriers to optimal AF treatment, particularly for initiating and maintaining long-term anticoagulation treatment from the perspective of HCPs working in primary care in a low- middle income area of Sao Paulo, Brazil. Our findings indicate that specific AF training is not currently available for all HCPs in PCUs although many felt there was a need for this. Knowledge and use of guidelines and risk scales are suboptimal; HCPs acknowledged that specific protocols and guidelines on AF management are needed. The lack of patient knowledge on the importance of continuous care as part of ideal management and on AF more generally is one of the main barriers faced by all HCPs. Better access to appointments with consultants in secondary care and availability of INR tests in primary care are needed to improve the multidisciplinary team currently required to provide optimal AF care in Sao Paulo. Though, whilst warfarin is widely available in PCUs, HCPs highlighted the need for NOACs to be freely available from the Brazilian Health National System which will reduce the need for INR tests.

Existing literature exploring the patient´s journey of AF care is from high-income countries (HICs) [8, 10], making our study the first to explore this topic in an LMIC setting. A 2017 qualitative systematic review by Dalmau et al. reported on patients’ and HCPs’ attitudes and perceptions of the risks, benefits and use of vitamin K antagonists such as warfarin [8]. A more recent (2020) qualitative meta-synthesis reported on HCPs views and experiences of prescribing anticoagulants for stroke prevention [10]. Some findings from these reviews were consistent with the present findings. For instance, working as a multidisciplinary team to manage AF patients was common in HICs and primary care HCPs often lacked experience and confidence with prescribing and controlling anticoagulation [8]. It was recognised that patients in HICs had a lack of information regarding the importance and impact of anticoagulation and often had misconceptions and misunderstandings about the medication [8]; this lack of patient knowledge was also perceived by the HCPs within our study. In concordance with our findings, the fear of causing haemorrhagic stroke and the use of risk assessment tools in HICs is also uncommon [10]. Although communication between primary and secondary care HCPs was a frequent issue in HICs [8, 10], this was not raised in the present study. Some other main barriers highlighted by HCPs in HICs were their perceived uncertainty in the decision-making of, and the need for evidence-based information on anticoagulation use, particularly in certain populations [8, 10]. This demonstrates the differences in what HCPs feel are important and more pertinent in LMICs compared to HICs; in the low-income area our study population is drawn from, the difficulty of monitoring AF in primary care is due to the lack of rudimentary infrastructure which is required to perform INR tests and offer timely medical consultations. A lack of clarity on safety in protocols [8] and inaccessible formats of guidelines [10] were issues raised in HICs, whereas our study highlights the need for the existence of a protocol/guideline. These barriers of AF care in HICs can and should be considered during the development of specialty training, guidelines and improvements to services in LMICs which our study highlighted there is the need for in the Butantan area of Sao Paulo.

In the present study, the lack of training for AF treatment and management in the primary care system was one of the main barriers to optimal AF care. Primary care in Brazil is mainly focused on prevention including vaccination, health promotion activities and identification and treatment of traditional cardiovascular risk factors such as diabetes, dyslipidaemia, smoking and hypertension [23]. The municipal health authority in primary care holds AF and anticoagulation as of relatively low importance compared to these traditional risk factors. Thus, resources have not been provided for structuring anticoagulation clinics nor developing a thorough training programme with evidence-based guidelines and protocols, which is reflected in the current findings. The primary care municipal health authority should include in its cardiovascular disease prevention programme the care of AF patients, initiating basic training for HCPs on AF care and provide the means for anticoagulation management to take place at the PCUs. The latter would include having locally available INR tests and more secondary care cardiology consultations. In contrast to the lack of guidelines and protocols in the PCUs, several evidence-based risk assessment tools exist although we observed low frequency and wide variation of their use. Stroke risk scales (e.g. CHA_2_DS_2_-VASc score) [24] and bleeding risk assessments (e.g. HAS-BLED) [25] are globally known and recommended in guidelines. They can easily be used in primary care as they are freely available online. These scores are the first step in risk assessment to inform appropriate prescription of anticoagulation for AF patients and could improve the care of these patients. This, together with local availability of prothrombin time (or implementation of anticoagulation clinics) for warfarin users, primary care HCP awareness campaigns to detect AF and assess AF stroke risk, could improve the AF detection and anticoagulation rates in Brazil.

Individualised treatment based on patient involvement in the decision-making process is of growing importance for all disease care pathways [8]. Providing the patient with information pertaining to AF and treatment, particularly warfarin, may improve adherence and patient outcomes [26, 27]. Similarly, including patients in the decision-making process for treatment plans may also improve patient adherence, outcomes and satisfaction [26, 27]. A mix of virtual and face-to-face education on AF with options to ask questions and hear patient testimonies have been noted as the patient’s preference for receiving information about AF [12]. A UK-based study [28] found significant improvements in TTR (time in therapeutic range) at 6-month follow-up after use of an educational intervention utilising an expert-patient DVD, educational booklet, self-monitoring diary and worksheet; however, the significant difference in improvements were not sustained at 12-month follow-up. The authors noted that repetition and reinforcement of educational messages over time may be needed to sustain clinical benefit [28]. The development of effective strategies to improve patients’ knowledge of their illness and treatment options, particularly in LMICs where data are lacking, should be a priority for future research.

Warfarin is available free of charge at the PCU pharmacies, unlike NOACs [29]. A previous Brazilian observational study of more than 200,000 primary care patients revealed an extremely low (2%) use of anticoagulation medication among AF patients [30], which has also been observed in other LMICs studies [31]. Low use of OACs could be due to a lack of INR tests in PCUs or lack of knowledge and experience from primary care physicians in managing patients with AF and assessing the risk of cardioembolic events, as the present study indicates. Other reasons could be due to patients’ fear of serious bleeding and limitations of vitamin K inhibitors such as interactions with alcohol, other medications and diet [30]. INR monitoring is not necessary with NOACs which are safer with respect to major and intracranial bleeding when compared with warfarin [32]; thus, the availability of NOACs could overcome existing barriers to treatment adherence and improve the ease and cost of AF management. Evidence suggests that NOACs are more cost-effective compared with warfarin [32, 33]; however, the feasibility and efficacy of using NOACs and their impact on treatment adherence should be investigated in the Brazilian setting.

Our study has some notable strengths. As the first study to present barriers and facilitators for optimal AF care in an LMIC, we included a large sample of primary HCPs from nearly all PCUs located in a low-middle income area of Sao Paulo, Brazil. Our use of a mixed-methods study design enables an in-depth interpretation of the contextual factors behind our findings. Additionally, we included a diverse group of HCPs including nurses, nurse technicians, community health agents and pharmacists whereas many studies performed in HICs have prioritised the perceptions of physicians [8–12]. Limitations of our study include the generalisability of our results. The population within our study which covered most PCUs in the western region of Sao Paulo may not reflect other locations within the city that have different healthcare resources nor populations in other LMICs. Additionally, the selection of only PCUs connected with the Universidade de Sao Paulo where medical students train may have new medical developments and innovations that differ from other PCUs. However, many of our results are in line with existing qualitative evidence from HICs indicating that some barriers and facilitators to AF care we highlight are shared globally. Our results will nonetheless serve as a comparative reference for future studies in other regions of Brazil and will provide an important and initial viewpoint on AF care in an LMIC. Lastly, whilst one of the quantitative questions related to barriers of care may have influenced responses given to the open-ended questions, the qualitative analysis identified facilitators as well as barriers and the barriers identified were different and more detailed than those listed in the close-ended question, providing context and a deeper understanding of the current system of care.

## Conclusions

The main barriers to optimal AF care identified in Brazilian PCUs were the lack of AF-specific training for HCPs, protocols and guidelines on AF management, patients’ understanding about AF, lack of available appointments with secondary care consultants, INR tests in the PCUs and availability of NOACs. AF care should be prioritised within current cardiovascular disease prevention programmes in Sao Paulo to enable the development and implementation of protocols, guidelines, training, necessary infrastructure to conduct tests and provide timely care to AF patients and availability of NOACs. These crucial points raised by the HCPs should guide future research and clinical practice in LMICs to achieve optimal AF care and management with a view to reduce the mortality and morbidity caused by AF-related strokes.

## Supplementary Information

**Additional file 1.** Quantitative questionnaire for healthcare professionals.

**Additional file 2.** Demographic characteristics of included participants.

**Additional file 3.** Questionnaire responses from healthcare professionals regarding the care and management for AF.

**Additional file 4.** Questionnaire responses from healthcare professionals regarding the treatment and monitoring for AF^a^.

**Additional file 5.** Supporting quotes from healthcare professional’s response to the open-ended questions.

## Data Availability

The datasets used and/or analysed during the current study are available from the corresponding author on reasonable request.

## Abbreviations

AF: Atrial fibrillation
LMICs: Low- and middle-income countries
OAC: Oral anticoagulation
HCPs: Healthcare professionals
PCUs: Primary care units
NIHR: National Institute for Health Research
FHS: Family health strategy
INR: International normalised ratio
NOACs: Novel oral anticoagulants
HICs: High-income countries
TTR: Time in therapeutic range
