# Supplementary material for: Health care professionals’ perceptions about atrial fibrillation care in the Brazilian public primary care system: a mixed-methods study
Source: BMC Cardiovasc Disord. 2022 Dec 22;22:559. doi: 10.1186/s12872-022-02927-9 (PMC9772592; doi:10.1186/s12872-022-02927-9)
Supplement: Supplementary file 1 — Additional file 1. Quantitative questionnaire for healthcare professionals. [file 12872_2022_2927_MOESM1_ESM.docx]

Additional file 1: Quantitative questionnaire for healthcare professionals

**Questionnaire for the primary care team**

**Version: DOCTOR**

| **CLINIC NAME / HEALTH UNIT** |  |
| --- | --- |
| **DOCTOR`S ID** |  |
| **DOCTOR´S NAME:** |  |

1. Do you follow patients with atrial fibrillation?

1.( ) Yes 🡪 CONTINUE

2.( ) No 3.( ) Don´t Know 🡪 FINISH

2. Do the patients with atrial fibrillation that you are following have an ECG that confirms the diagnosis of atrial fibrillation?

1.( ) Yes 🡪 CONTINUE

2.( ) No 3.( ) Don´t Know 🡪 JUMP TO Q4

3. In cases of atrial fibrillation confirmed by ECG, the prescribed treatment includes:

1.( ) Aspirin

2.( ) Warfarin

3. ( ) Other anticoagulants. Specify the medicine used

____________________________________________________________________

4. Do you follow any consensus / guideline for the treatment of atrial fibrillation?

1.( ) Yes 🡪 CONTINUE

2.( ) No 3.( ) Don´t Know 🡪 JUMP TO Q6

5. Specify the consensus / guideline used:

____________________________________________________________________

6. Do you use any risk scale before deciding on the type of treatment?

1.( ) Yes 🡪 CONTINUE

2.( ) No 3.( ) Don´t Know🡪 JUMP TO Q8

7. What´s the risk scale?

_____________________________________________________________________________

8. Who participates in the decision making to start anticoagulation?

1.( ) Only the doctor from the secondary care (for example, HU internal medicine outpatient clinic or cardiologist in Peri-Peri)

2.( ) The doctor from the secondary care (for example, HU internal medicine clinic or Peri-Peri cardiologist) and the doctor from the Family Health Strategy (FHS)

3.( ) Only the FHS doctor

4.( ) The FHS doctor with the nurse

5.( ) The FHS doctor, the nurse and the nursing technician

6.( ) The FHS doctor, the nurse, the nursing technician and the community agent

7.( ) The FHS doctor, the nurse, the nursing technician and the community agent and the pharmacist

9. Do you refer your patient with atrial fibrillation to the Peri-Peri Specialty Clinic to collect coagulograms?

1. ( ) Yes 🡪JUMP TO Q11

2.( ) No 3.( ) Don´t Know 🡪 CONTINUE

10. Do you advise your atrial fibrillation patient to collect the coagulogram in the unit itself?

1.( ) Yes 2.( ) No 3.( ) Don´t Know

11. What are the main barriers in monitoring patients with atrial fibrillation with warfarin (or other oral anticoagulant that requires INR control) in the Unit?

1.( ) Difficulty in collecting the coagulogram

2.( ) Delays in releasing results

3.( ) Difficulty of the patient to understand how to take the medication

4. ( ) Fear of severe bleeding

5.( ) Difficulty obtaining medications at the unit

6.( ) Patient´s low adherence to the treatment

7.( ) Interaction of warfarin with other drugs

8. ( ) Interaction of warfarin with diet

12. Are there patients with atrial fibrillation using anticoagulants other than warfarin?

1.( ) Yes 2.( ) No 3.( ) Don´t Know

13. Have you had any specific training in the treatment of atrial fibrillation since you started working at the unit ?

1.( ) Yes 🡪 CONTINUE

2.( ) No 3.( ) Don´t Know 🡪 JUMP TO Q15

14. What kind of training?

___________________________________________________________________________

15. Do you have residency in Family and Community Medicine?

1.( ) Yes 🡪 CONTINUE

2.( ) No 3.( )Don´t Know 🡪 JUMP TO Q17

16. What year will the residency end: ______________________________

17. Do you have the title of specialist in Family and Community Medicine?

1.( ) Yes 🡪 CONTINUE

2.( ) No 3.( ) Don´t Know 🡪 FINISH

18. What year did you receive the title:

______________________________

Initial open-ended questions

1) Who are the patients with AF that you follow?

2) How is AF diagnosed and followed-up?

3) In your opinion, what are the positive and negative points around caring for and monitoring AF patients?

4) How is the interaction between AF patients and you and other team members at this PCU?

5) In your opinion, how could the interaction between AF patients and you and the other team members be improved at this PCU?
