# Supplementary material for: Health care professionals’ perceptions about atrial fibrillation care in the Brazilian public primary care system: a mixed-methods study
Source: BMC Cardiovasc Disord. 2022 Dec 22;22:559. doi: 10.1186/s12872-022-02927-9 (PMC9772592; doi:10.1186/s12872-022-02927-9)
Supplement: Supplementary file 2 — Additional file 2. Demographic characteristics of included participants. [file 12872_2022_2927_MOESM2_ESM.docx]

Additional file 2. Demographic characteristics of included participants

|  | Managers  (n=9) | Family practice doctors (n=13) | General clinicians (n=9) | Nurses (n=16) | Nurse technicians (n=18) | Community health agents (n=29) | Pharmacists  (n=13) | Total  (n=107) |
| --- | --- | --- | --- | --- | --- | --- | --- | --- |
| **Age** |  |  |  |  |  |  |  |  |
| Mean (SD) | 41.3 (3.9) | 35.0 (5.8) | 34.6 (6.3) | 40.4 (7.5) | 40.9 (9.2) | 38.5 (10.3) | 32.1 (6.8) | 37.9 (8.5) |
| **Sex** |  |  |  |  |  |  |  |  |
| Male | 2 (22) | 3 (23) | 5 (56) | 3 (19) | 1 (6) | 2 (7) | 2 (15) | 18 (17) |
| Female | 7 (78) | 10 (77) | 4 (44) | 13 (81) | 17 (94) | 27 (93) | 11 (85) | 89 (83) |
| **Education** |  |  |  |  |  |  |  |  |
| College | 9 (100) | 13 (100) | 9 (100) | 16 (100) | 0 (0) | 0 (0) | 13 (100) | 60 (56) |
| High school | 0 (0) | 0 (0) | 0 (0) | 0 (0) | 18 (100) | 29 (100) | 0 (0) | 47 (44) |
| **Duration of professional experience** |  |  |  |  |  |  |  |  |
| Mean years (SD) | 16.9 (6.4) | 7.5 (3.9) | 6.9 (6.0) | 11.3 (7.3) | 9.6 (6.3) | 7.6 (3.0) | 9.0 (7.2) | 9.5 (6.1) |
