# Supplementary material for: Health care professionals’ perceptions about atrial fibrillation care in the Brazilian public primary care system: a mixed-methods study
Source: BMC Cardiovasc Disord. 2022 Dec 22;22:559. doi: 10.1186/s12872-022-02927-9 (PMC9772592; doi:10.1186/s12872-022-02927-9)
Supplement: Supplementary file 3 — Additional file 3. Questionnaire responses from healthcare professionals regarding the care and management for AF. [file 12872_2022_2927_MOESM3_ESM.docx]

Additional file 3. Questionnaire responses from healthcare professionals regarding the care and management for AF

|  | **Family practice doctors**  **(n=13)** | **General clinicians (n=9)** | **Nurses**  **(n=16)** | **Nurse technicians**  **(n=18)** | **Community health agents**  **(n=29)** | **Pharmacists (n=13)** | **Total sample**  **(n=98)** |
| --- | --- | --- | --- | --- | --- | --- | --- |
| **Do the patients with AF that you are following have an ECG that confirms the diagnosis of AF?** |  |  |  |  |  |  |  |
| Yes | 13 (100) | 8 (89) | 16 (100) | 18 (100) | 24 (83) | -- | 79/85 (93) |
| No | 0 | 1 (11) | 0 | 0 | 2 (7) | -- | 3/85 (4) |
| Don´t know | 0 | 0 | 0 | 0 | 3 (10) | -- | 3/85 (4) |
| **In cases of AF confirmed by ECG, the prescribed treatment included what medications? ^a^** |  |  |  |  |  |  |  |
| Warfarin | 12 (92) | 8 (89) | 11 (69) | 7 (39) | 17 (59) | 11 (85) | 66 (67) |
| Aspirin | 6 (46) | 4 (44) | 12 (75) | 10 (56) | 19 (66) | 5 (38) | 56 (57) |
| NOACs | 3 (23) | 5 (56) | 3 (19) | 5 (28) | 1 (3) | 0 | 17 (17) |
| Others | 2 (15) | 0 | 2 (13) | 4 (22) | 1(3) | 0 | 9 (9) |
| Don´t know | 0 | 0 | 0 | 1 (6) | 3 (10) | 0 | 4 (4) |
| **Do you follow any consensus / guideline for the treatment of AF?** |  |  |  |  |  |  |  |
| Yes | 6 (46) | 3 (33) | 4 (25) | 6 (33) | 4 (14) | 4 (31) | 27/98 (28) |
| No | 6 (46) | 5 (56) | 9 (56) | 4 (22) | 11 (38) | 8 (62) | 43/98 (44) |
| Don´t know | 1 (8) | 1 (11) | 3 (19) | 8 (44) | 14 (48) | 1 (8) | 28/98 (29) |
| **Do you use any risk scale before deciding on the type of treatment?** |  |  |  |  |  |  |  |
| Yes | 9 (69) | 4 (44) | 8 (50) | 7 (39) | 7 (24) | 4 (31) | 39/98 (40) |
| No | 4 (31) | 3 (33) | 4 (25) | 6 (33) | 7 (24) | 8 (62) | 32/98 (33) |
| Don´t know | 0 | 2 (22) | 4 (25) | 5 (28) | 15 (52) | 1 (8) | 27/98 (28) |
| **Have you had any specific training in the treatment of AF since you started working at the unit?** |  |  |  |  |  |  |  |
| Yes | 0 | 2 (22) | 3 (19) | 3 (17) | 0 | 1 (8) | 9/98 (9) |
| No | 13 (100) | 7 (78) | 13 (81) | 15 (83) | 28 (97) | 12 (92) | 88/98 (90) |
| Don´t know | 0 | 0 | 0 | 0 | 1 (3) | 0 | 1/98 (1) |

-- This question was not relevant for this particular HCP and therefore data has been omitted.

^a^ Multiple-choice question; proportions may not add up to 100%.
