# Supplementary material for: Health care professionals’ perceptions about atrial fibrillation care in the Brazilian public primary care system: a mixed-methods study
Source: BMC Cardiovasc Disord. 2022 Dec 22;22:559. doi: 10.1186/s12872-022-02927-9 (PMC9772592; doi:10.1186/s12872-022-02927-9)
Supplement: Supplementary file 4 — Additional file 4. Questionnaire responses from healthcare professionals regarding the treatment and monitoring for AFa. [file 12872_2022_2927_MOESM4_ESM.docx]

Additional file 4. Questionnaire responses from healthcare professionals regarding the treatment and monitoring for AF ^a^

|  | **Family practice doctors**  **(n=12)** | **General clinicians**  **(n=8)** | **Nurses**  **(n=11)** | **Nurses technicians**  **(n=7)** | **Community health agents**  **(n=17)** | **Pharmacists (n=11)** | **Total sample**  **(N=66)** |
| --- | --- | --- | --- | --- | --- | --- | --- |
| **Do you refer your AF patients have their INR tests in secondary care (cardiology) to?** |  |  |  |  |  |  |  |
| Yes | 11 (92) | 8 (100) | 10 (91) | 7 (100) | 13 (76) | 4 (36) | 53 (80) |
| No | 0 | 0 | 0 | 0 | 2 (12) | 5 (45) | 7 (11) |
| Don´t know | 1 (8) | 0 | 1 (9) | 0 | 2 (12) | 2 (18) | 6 (9) |
| **Do you advise your AF patient to have their INR tests in the unit itself?** |  |  |  |  |  |  |  |
| Yes | 0 | 0 | 0 | 0 | 2 (12) | 1 (9) | 3 (5) |
| No | 11 (92) | 8 (100) | 10 (91) | 7 (100) | 12 (71) | 7 (64) | 55 (83) |
| Don´t know | 1 (8) | 0 | 1 (9) | 0 | 3 (18) | 3 (27) | 8 (12) |
| **What are the main barriers in monitoring patients with AF under warfarin use (or other oral anticoagulant that requires INR control) in the Unit? ^b^** |  |  |  |  |  |  |  |
| Difficulty of the patient to understand how to take the medication | 6 (50) | 5 (63) | 7 (64) | 7 (100) | 9 (53) | 7 (64) | 41 (62) |
| Fear of severe bleeding | 9 (75) | 7 (88) | 8 (73) | 4 (57) | 8 (47) | 2 (18) | 38 (58) |
| Interaction of warfarin with diet | 7 (58) | 6 (75) | 4 (36) | 3 (43) | 10 (59) | 5 (45) | 35 (53) |
| Interaction of warfarin with other drugs | 7 (58) | 5 (63) | 4 (36) | 2 (29) | 11(65) | 5 (45) | 34 (52) |
| Difficulty of collecting coagulogram | 9 (75) | 3 (38) | 5 (45) | 5 (71) | 9 (53) | 1 (9) | 32 (48) |
| Delays in releasing results | 10 (83) | 3 (38) | 4 (36) | 2 (29) | 7 (41) | 1 (9) | 27 (41) |
| Low patient adherence to treatment | 2 (17) | 3 (38) | 3 (27) | 5 (71) | 8 (47) | 7 (64) | 28 (42) |
| Difficulty of obtaining medications at primary care | 0 | 1 (13) | 2 (18) | 3 (43) | 7 (41) | 1 (9) | 14 (21) |
| Do not bring anticoagulation control card | 0 | 0 | 0 | 0 | 0 | 1 (9) | 1 (2) |
| Don´t know | 0 | 0 | 0 | 0 | 0 | 0 | 0 |

^a^ Only HCPs who said they prescribe or supervise Warfarin use were asked these questions.

^b^ Multiple-choice question; proportions may not add up to 100%.
