# Supplementary material for: Health care professionals’ perceptions about atrial fibrillation care in the Brazilian public primary care system: a mixed-methods study
Source: BMC Cardiovasc Disord. 2022 Dec 22;22:559. doi: 10.1186/s12872-022-02927-9 (PMC9772592; doi:10.1186/s12872-022-02927-9)
Supplement: Supplementary file 5 — Additional file 5. Supporting quotes from healthcare professional’s response to the open-ended questions. [file 12872_2022_2927_MOESM5_ESM.docx]

Additional file 5. Supporting quotes from healthcare professional’s response to the open-ended questions

| **Question 1. AF patients attended by HCPs** **Who are these patients with AF that you monitor (follow)?** | | | |
| --- | --- | --- | --- |
| **Sociodemographic characteristics** | | | “They are old men…” (Physician) |
| **Health and lifestyle habits** | | | “They are … with comorbidities, who already had a heart attack.” (Physician)  “They are smokers, sedentary” (Nurse)  “They have a bad diet, rich in fats.” (Nurse technician) |
| **Question 2. How is the AF care/follow-up?** | | | |
| **Receiving a diagnosis of AF** | | Medical visit in the PCUs due to patient feeling unwell | “Some patients arrive feeling sick, so they go with the doctor, we do the electro and they are referred to the cardiologist.” (Nurse)  “Many patients arrive at the unit feeling unwell and are examined by the doctor who, after the electrocardiogram, sends them to the reference hospital in the region.” (Nurse Technician) |
|  |  | Patients arrive at the PCUs with the AF already diagnosis | “Patients arrive here diagnosed and with a prescription ... they only come to change the prescription with the doctor and get the medicine.” (Manager)  “Patients are already diagnosed here, so we just need to follow up, advise on medications and monitor other comorbidities.” (Physician) |
|  |  | Regular visits and consultations | “Patients are identified at home during the monthly visits” (Nurse)  “They arrive by the team on the home visit and the agent makes an appointment with the doctor and, if necessary, the doctor collects the electrocardiogram at the unit and forwards them to do the INR. “(Manager)  “Some patients are identified with AF in the routine consultation.” (Physician)  “Some patients are identified during routine exams and are referred to the cardiologist.” (Nurse Technician) |
| **Follow-up after AF diagnosis** | | Follow-up before stabilisation | “In some cases, the return is made 15 days before stabilization.” (Physician)  “In some cases, we evaluate every fortnight, doing a double follow-up, as we have greater availability here in the units.” (Physician)  “In some cases, monitoring is done weekly.” (Physician)  “When the patient returns from the cardiologist with the exams, we will evaluate weekly.” (Physician) |
|  |  | Follow-up after stabilisation | “The return is made 1 to 2 months after stabilization.” (Physician)  “Some patients are already diagnosed by the cardiologist, so we follow up every 1 or 2 months.” (Physician)  “The follow-up is done every 3/6 months.” (Physician)  “Some patients, after stabilized, they return every 3 months to adjust the medication.” (Physician) |
|  |  | Communication and referral between PCUs, secondary care and tertiary care settings | “The patient is referred for reassessment with a cardiologist in [secondary care setting] or [tertiary care setting].” (Physician)  “When the patient arrives in the emergency room, we stabilize and send them to the specialist.” (Physician)  “The patient continues the treatment with a cardiologist if it is severe.” (Physician)  “After the electrocardiogram, I forward them to the cardiologist referenced in the region.” (Physician)  “Sometimes the patients are referred to the cardiologist to set the anticoagulant.” (Physician)  “The patients who are diagnosed in the unit, we ask for an electrocardiogram, forward it to the cardiologist.” (Physician)  “The doctor performs the electrocardiogram, forwards the patient to the cardiologist and then they start the anticoagulant together.” (Nurse Technician)  “After seeing the cardiologist, they return to the PCUs to periodically adjust the medication.” (Physician) |
| **Barriers and facilitators for AF care in PCUs** | | | |
| **Access to care** | Access to appointments | | Facilitators:  “The access to medical care is good, consultations are available.” (Nurse)  “There is good availability for consultation with doctors.” (Manager) |
|  |  |  | Barriers:  “The professionals are overloaded to care for patients with AF.” (Physician)  “The team is unable to proceed with the monitoring, there is not enough team, the patient with AF has to be referred to secondary care.” (Pharmacist)  “We lack professionals specialized in AF.” (Community Health Agent)  “There are no ambulances available to take patients to a hospital in emergency cases.” (Nurse)  “Many patients have limited mobility to go to the cardiologist.” (Physician)  “It is very difficult to get an appointment with a cardiologist.” (Physician)  “The patient has to wait a long time to get an appointment with the cardiologist.” (Physician)  “There is little availability of appointments with the cardiologist so monitoring is difficult.” (Physician) |
|  | Access to equipment and tests | | Facilitators:  “As soon as the doctor diagnoses the presence of AF, he asks for the electrocardiogram and the patient does it right away here in the unit.” (Manager)  “We have the electrocardiogram to make the diagnosis” (Nurse Technician) |
|  |  |  | Barriers:  “There is no structure to collect the INR at the PCUs” (Physician)  “To perform the INR is often difficult because the patient has to travel to the Peri Peri unit (Secondary care)” (Physician)  “The negative is that here you can't do the INR! They have to do outside of the unit.” (Physician)  “There is no structure to collect INR at the PCUs. This makes the treatment difficult” (Physician)  “The INR exam should be done in the units to facilitate the care of patients” (Physician)  “[PCUs] should have INR collection in the unit itself because patients have financial and locomotion difficulties to go to the [secondary care].” (Nurse Technician) |
|  | Access to medication | | Facilitators:    “The necessary medications are available. Warfarin is never lacking.” (Pharmacist)  “Here in this unit, warfarin is never lacking. “(Physician) |
|  |  |  | Barriers:  “There is a lack of better and more modern drugs for the treatment of AF.” (Physician)  “Some specific drugs are missing in the public system (Pharmacies from PCUs).” (Manager)  “We should have other better drug options, like Xarelto, which would make it much easier, as it would do away with the control and consultations with the specialist.” (Physician) |
| **HCP and patient roles** | HCPs relationship with their patients | | Facilitators:  “The team has a good link with the patients, has a good relationship with them and knows the patient's history” (Manager)  “The professionals are committed; the community has a good link with the team “(Physician)  “The community agent is very close to the patients.” (Pharmacist)  “The doctors are dedicated to their patients.” (Nurse Technician)  “We have a good interaction with the patient, we are able to assist and accompany the patient” (Community Health Agent)  “We have well-prepared teams, the professionals are close to the patients, they have a good relationship with the patients and their families.” (Nurse Technician)  “The great positive point of this unit is a competence and attention, dedication and proximity of the professionals of the teams with the patients.” (Manager) |
|  | Patient’s adherence to HCP’s advice | | Facilitators:  “The patients are receptive and caring.” (Nurse Technician)  “Patients usually follow the instructions from their doctors to take the medication correctly.” (Physician) |
|  |  |  | Barriers:  “Some patients are more resistant and do not accept the medications.” (Community Agent)  “Some patients deny the problem and are more resistant.” (Nurse Technician)  “The patients need to be informed about what AF is and prepared to follow the treatment” (Physician)    “The patient needs to understand the disease and the importance of the treatment.” (Pharmacist) |
| **Organisation / system level role** | Lack of private space | | Barriers:  “Insufficient and an adequate structure is lacking to care [for] patients with AF.” (Physician)  “There is a lack of adequate physical space … in AF care. “(Nurse)  “There is a lack of adequate structure for attending the patient with AF.” (Nurse Technician)  “It should have an exclusive private room for the care of patients with AF.” (Nurse Technician)  “The emergency room is too small and poorly equipped to care for the patient with AF and has no privacy.” (Nurse) |
|  | Lack of specific training | | Barriers:  “There is a lack of specific training for the care of patients with AF “(Manager)  “It would be necessary to have specific training of the staff to care for patients with AF...” (Physician)  “There is a lack of … specific training in AF care.” (Nurse)  “The teams do not have a specific focus on AF.” (Physician)  “The teams are not trained to attend AF.” (Nurse)  “We need more training for the entire team to better manage the patient with AF.” (Nurse)  “We need to have specific courses and training on FA.” (Physician)  “There is a lack of training for nursing teams to feel more secure to manage patients with AF.” (Nurse) |
|  | Lack of guidelines and protocols | | Barriers:  “There is a lack of … protocols for AF care.” (Nurse technician)  “We need to have adequate protocols and guidelines to care for the patient with AF.” (Nurse Technician) |
|  | Delay in INR results | | Barriers:  “The test results take a long time to be ready” (Physician)  “The INR exam results take more than 1 week to arrive! We would need to have the results on the same day!” (Physician) |
